# The Significance of Sleep Disorders in Post-myocardial Infarction Depression

**DOI:** 10.7759/cureus.30899

**Published:** 2022-10-31

**Authors:** Sai Dheeraj Gutlapalli, Jingxiong Pu, Maheen F Zaidi, Maithily Patel, Lakshmi Malvika Atluri, Natalie A Gonzalez, Navya Sakhamuri, Sreekartthik Athiyaman, Bhawna Randhi, Sai Sri Penumetcha

**Affiliations:** 1 Internal Medicine, California Institute of Behavioral Neurosciences & Psychology, Fairfield, USA; 2 Psychiatry and Behavioral Sciences, California Institute of Behavioral Neurosciences & Psychology, Fairfield, USA; 3 Family Medicine, California Institute of Behavioral Neurosciences & Psychology, Fairfield, USA; 4 Surgery, California Institute of Behavioral Neurosciences & Psychology, Fairfield, USA; 5 Pediatrics, California Institute of Behavioral Neurosciences & Psychology, Fairfield, USA; 6 Medicine, NRI Medical College, Chinakakani, IND; 7 Medicine, California Institute of Behavioral Neurosciences & Psychology, Fairfield, USA; 8 General Medicine, California Institute of Behavioral Neurosciences & Psychology, Fairfield, USA; 9 General Medicine, Chalmeda Anand Rao Institute of Medical Sciences, Karimnagar, IND

**Keywords:** sleep disorders, myocardial infarction, sleep apnea syndrome, major depression disorder, post-myocardial infarction

## Abstract

Sleep disorders are highly prevalent and often missed comorbidities in patients with cardiovascular disease (CVD), especially important in patients with depression associated with post-myocardial infarction (MI). Proactive screening and targeted treatment for sleep disorders are essential to reduce the morbidity and mortality associated with CVDs and MI in particular. We have reviewed all relevant information up to July 2022 regarding sleep disorders in CVD with a focus on post-MI depression and gathered around 350 articles in our research and narrowed it down to 31 articles. The database used was PubMed, and the keywords used were obstructive sleep apnea, sleep disorders, sleep-disordered breathing, major depression, and post-myocardial infarction. We have concluded from the available literature that there is a significant overlap between the etiologies and pathological mechanisms between conditions such as diabetes, obesity, and other comorbidities associated with both sleep disorders and CVD. Treatment such as psychotherapy and pharmacotherapy should be tailored according to the specific needs of the patients. Targeted treatment for sleep disorders has been shown to improve multiple factors and comorbidities associated with prognosis post-MI, including improvement in quality of life and significantly reduced short-term and long-term mortality. The incidence and prevalence of depression post-MI can be significantly reduced by focusing on the treatment of any underlying sleep disorders. We encourage larger-scale observational and interventional studies regarding the quality of sleep post-MI.

## Introduction and background

The National Commission on Sleep Disorders estimated that sleep apnea might be directly or indirectly responsible for almost 38,000 cardiac deaths every year in America [1]. Cardiovascular disease (CVD) and major depression (MD) are also closely associated. Depression may trigger or precipitate myocardial infarction (MI) and vice versa [2]. Everyday around 2,500 people with CVD in the United States die of cardiac complications [3]. Coronary artery disease (CAD) is linked to the incidence of anxiety, panic attacks, and major depressive disorder (MDD) [4-6]. Factors such as stress, MD, anxiety, and sleep disorders are directly linked to a worse prognosis with pre-existing CAD [7]. Major depression is independently linked to CAD and 20% have MD while depressive symptoms are prevalent in a majority of CAD patients, thus increasing morbidity and mortality [8,9].

Psychological illness is linked to medication non-compliance and reduced treatment follow-up, higher rates of hospitalizations, reduced overall function, and high mortality in heart diseases [10]. Even in patients without a history of CVD, there is an increased risk of arrhythmia and sudden cardiac death (SCD) associated with depression, and the risk is significantly increased in association with depression [7,8]. Clinical evidence supports that cognitive behavior therapy (CBT), and psychotherapy along with pharmacotherapy for the treatment of depressed patients with CVD and sleep disorders have been shown to decrease the efficacy of one or both [11,12].

Our research focuses on the relevance of sleep disorders in post-MI depression. In view of the immense global burden of MD and comorbid sleep disorders in CVD patients. We try to understand the effect of sleep disorders on post-MI depression regarding morbidity and mortality in the short and long term. The relevant data for this literature review was gathered from the PubMed database. The following five Keywords were used: “Major Depression,” “Post-Myocardial Infarction,” “Sleep disorders,” “Obstructive sleep apnea,” and “sleep disordered breathing.” The search was performed using Medical Subject Headings (MeSH) strategy. We manually screened and included all relevant articles we could find from inception till July 18th, 2022. All data were sourced from PubMed.

## Review

Heart disease and depression

Over the course of the next decade, psychiatric illness and CVD will account for the majority of disease burden and mortality in the world. CAD and depression specifically are the major issues leading to long-term disability [6,8,9]. Patients with pre-existing CVD have a higher risk and frequency of psychiatric issues such as anxiety and depression as well as poor prognosis after cardiac events, while patients with mental health issues are at an increased risk of new-onset CVD. MDD and MI can mutually trigger or aggravate the other condition. Low mood is seen in half of CVD patients while one in five has MDD [2,4,5,7,8]. Two-thirds of MI patients have an associated psychiatric illness [6]. MD is observed in half of the patients with CAD in their lifetime [6]. Almost one in four patients after coronary artery bypass graft (CABG) surgery are reported to have MD [6]. Patients with a previous history of MD have four times the risk of MI compared to those without previous depression, and a history of MI is an independent risk factor for new-onset major depression in the hospital [6]. Psychiatric illness is associated with a three to four times higher rate of medication non-compliance, significantly higher risk of diabetes, malnutrition or obesity, smoking and alcohol/substance abuse, sleep disorders, and higher rates of hospitalizations and mortality in patients with heart disease [6,7,10,11].

Significantly higher levels of inflammation is associated with depression and worsened prognosis in CVD associated with adverse cardiovascular remodeling leading to cardiac dysfunction [10]. MD is linked to autonomic dysfunction which leads to a reduction in heart rate variability (HRV) leading to adverse cardiac remodeling, endothelial and platelet dysfunction, elevated C-reactive protein (CRP), reduced flow-mediated dilation in vessels leading to acceleration of atherosclerotic plaque formation, and CAD progression [6,10]. Studies have shown higher rates of arrhythmias, recurrent MI, angina, and congestive heart failure (CHF) during the initial hospital admission and significantly higher rates of readmissions in depressed patients compared to controls. Morbidity and mortality were doubled compared to controls [6,9].

Depression is a risk predictor for CHF hospitalization rate and post-MI mortality, a prodrome of minor or borderline depression sometimes precedes MI by four to five years [6,9]. Patients with specific personalities such as type D personality have a higher frequency of anxiety and depression [7]. Even in patients without any evidence of CVD, risk of arrhythmia and SCD is higher when associated with depression [8]. In the United States, more than eight million people have been diagnosed with heart failure (HF), by 2020 and the associated risk of mortality is 50% within the next five years after diagnosis. Among these patients, almost four million have comorbid MD [7,8,10].

According to the American Heart Association, the risk of MDD is almost three times higher in patients with acute coronary syndrome (ACS) when compared to the general population, and the risk of ACS is higher in patients with MDD and neurotransmitter dysregulation like serotonin, dopamine, and norepinephrine. Sympathetic/parasympathetic system disequilibrium in ACS and MDD further worsens the prognosis in patients [8,9]. Baroreflex sensitivity (BRS) and HRV are important tools to thoroughly understand these mechanisms [9]. A higher HRV is considered protective toward the development of HF, MI, and arrhythmias [8]. Cardiovagal activity is the mechanism that dictates HRV, and 70% of the cardiovagal tone is controlled by the arterial baroreflex system [9]. The incidence and prevalence of factors such as a response to acute stress, hypertension (HTN), cardiomyopathy, CHF, MI, ventricular fibrillation after MI, and SCD are associated with reduced BRS and HRV [9].

In the United States, almost seven million people and 15 million people in Europe are currently suffering from CHF. Depression more than triples the risk of CHF and doubles the all-cause mortality when compared to the general population and impairs response to treatment [11]. In the United States, almost 15% of the elderly population is currently taking anti-depressant pharmacotherapy [13]. Globally, one in five people is affected by depression in their lifetime [14]. Each year a million Americans have ACS, and half of them were previously depressed. ACS is broadly used to refer to unstable angina (UA) and MI [15]. Acute anxiety is seen in half of the patients post-MI, and persistent anxiety symptoms are also seen in some patients for almost two years [15]. Patients with CAD have a 40% higher incidence of anxiety than patients without CAD [15]. Patients with MD and MI in-hospital are reported to be depressed for more than a month before the acute event in 95% of the cases, and 60% of patients were depressed for six months or more. Untreated MD post-MI persists for years without decreasing in severity [15].

Elevated levels of corticotropin-releasing factor (CRF) in cerebrospinal fluid (CSF) are observed in patients with depression leading to a rise in corticosteroid levels, accelerated atherosclerosis, HTN, hypertriglyceridemia, and hypercholesterolemia [6]. QT variability increases and HRV decreases in MD leading to an elevated risk of arrhythmia, low HRV, and increased risk of overall mortality post-MI [6,8]. Childhood stress is also linked to the development of CVD in adulthood, and social support is preventive for the development of CVD [7]. Higher stress levels in males during and post-MI were associated with four to five times higher mortality rate [16]. Half of the patients with CVD also have cognitive deficits, especially patients with severe cardiac insufficiency or history of CABG. Further, the risk of dementia is also twice as high in patients with HF [7]. Patients with psychiatric illness and CVDs should be screened for comorbid dementia [7,14].

Acute psychological stress, anxiety, and depression are known triggers for new and recurrent arrhythmias, ventricular tachycardia (VT), stress-induced cardiomyopathy, and ACS [7]. Functional cardiac issues such as chest pain and palpitations without a known organic cause confuse clinicians as they are primarily psychogenic in nature in almost 15-20% of cases, and more than half of functional heart problems occur in patients with psychiatric illnesses [7]. The level of cardiac dysfunction and likelihood of progression to CHF is correlated with post-MI depression. Moreover, higher severity of depression is linked to a reduction in left ventricular ejection fraction (LVEF) 3-12 months post-MI, and LVEF <30% post-MI has a more than four times higher odds ratio for incidence of depression compared to patients with LVEF >60% (preserved EF) [11]. The single most efficient treatment for MD is electroconvulsive therapy (ECT) which has a response rate that is four times higher compared to other forms of treatment. Cardiac-modified ECT protocol is especially useful in patients with CVD [11].

Around $50 billion USD is spent on expenses related to CHF alone, and the cost of care is 30% higher for patients with associated psychiatric illnesses in the United States. The cost of care is 1% of the overall gross domestic product (GDP) in Europe for the treatment of depression [9,11]. Depression is an independent risk factor for incidence of MI and CVD in the long term based on the Johns Hopkins precursor Study. Depression increases risk of CVD by 60% [15]. Studies show pre and post-MI depression significantly worsens healthcare outcomes with higher incidence of recurrent adverse cardiac events in-hospital and mortality by as much as 2.6 times at the one-year follow-up with double the rate of recurrent MI when compared to non-depressed patients [14]. Treatment-resistant post-MI depression was associated with the highest risk of adverse outcomes in the long term [15]. Patients with MD had readmission rates as high as 80% over the one-year follow-up post-MI [17]. Some studies have shown that severe anxiety is a more dangerous risk factor for heart disease than MD. Anxiety is associated with higher short and long-term morbidity and mortality following acute cardiac events and post-MI [14]. In patients with HF suffering from post-MI depression, there was reduced motivation to follow a healthy lifestyle and complete cardiac rehabilitation program [10,14].

Post-MI depression increases the risk of all-cause mortality by three times in CVD patients [3]. Half an hour of routine exercise has similar effectiveness as pharmacotherapy against depression and brings down all-cause mortality by 25% in CVD. Post-MI depression during cardiac rehabilitation can be effectively managed by a combination of psychotherapy, anti-depressants, and exercise with a 75% reduction in all-cause mortality compared to patients who do not exercise. Regular exercise alone reduced the rates of non-fatal reinfarction, adverse events, and overall mortality by 50% post-MI at the two-year follow-up [6,7,14,15].

CBT is effective in treating depression and anxiety in HF with improved quality of life post-MI. Anti-depressant pharmacotherapy is more effective than CBT in patients with CVDs, especially CAD. Globally, 30% of deaths are associated with heart disease, and depression is prevalent in 30% of CAD and HF patients [6,10,15,18]. Depression during and post-MI worsens the prognosis of patients in the hospital and increases long-term cardiac morbidity and mortality. Depression causes noradrenergic system hyperactivity and serotonergic system dysregulation leading to poor outcomes by means of vascular stress induced by catecholamines on blood vessels, impaired platelet function, increased inflammation, lower HRV, endothelial dysfunction, and adverse cardiac remodeling, worsening prognosis in CAD and MI [6,10,15,18]. Studies focused on CBT and religious cognitive behaviour therapy (RCBT) over 12 sessions of therapy observed that RCBT was slightly more effective in religiously inclined people, but both CBT and RCBT were effective in treating high-risk patients with CVD and comorbid MDD [19].

Prevalence of sleep disorders in cardiovascular disease

It is estimated that sleep apnea is responsible for more than 100 cardiac deaths each day in America [1]. The risk factors for sleep disorders are age >35 years, body mass index (BMI) ≥25 kg/m^2^, alcoholism, higher Epworth sleepiness scale (ESS), higher oxygen desaturation index (ODI), higher nocturnal oxygen desaturation (NOD), and higher mean apnea duration [1]. CAD and insomnia may have a causal relationship [20]. A history of depression, female gender, and distress during ACS are key to identifying patients at higher risk of developing persistent sleep disorders [20]. About 29-67% of patients with insomnia have associated obstructive sleep apnea (OSA) [20].

**Figure 1:**
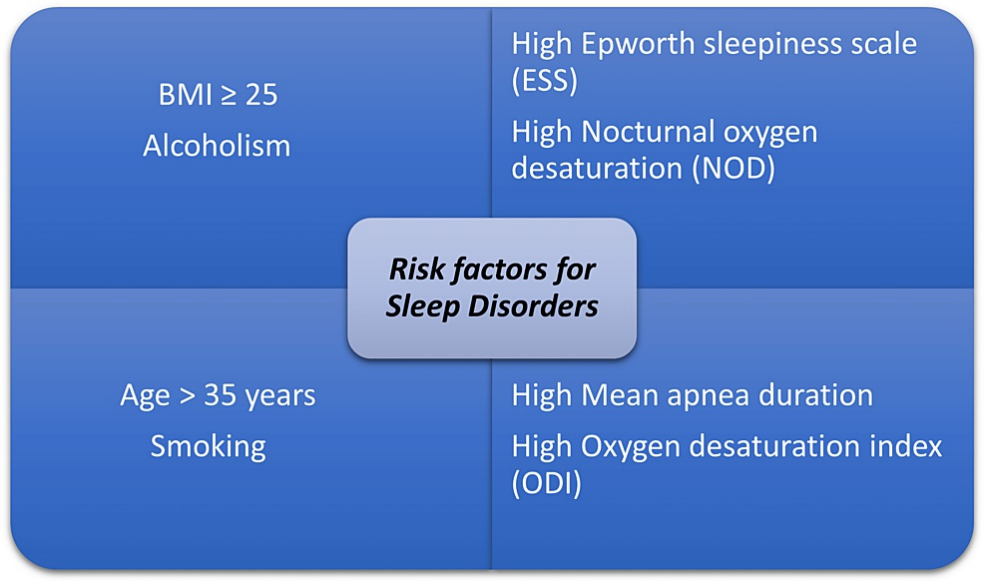
The risk factors for sleep disorders.

In a study done in Oman, post-MI patients had poor sleep quality, affecting more than 60% of patients in the study [21]. Sleep disturbances were common and affected latency and duration. In patients with associated mild depression, the potential for transition to MD was extremely high [21]. In post-MI patients, insomnia had a prevalence of more than 35%, and around 65% of the patients with insomnia post-MI had depressive symptoms. These issues increased the illness burden during cardiac rehabilitation [22]. In a study on acute post-MI sleep disorders, insomnia was observed in 27% of patients and OSA risk was higher in 41% of patients. Higher levels of fibrinogen and lower levels of norepinephrine were associated with more severe symptoms of OSA and insomnia [23]. Up to 50% of OSA patients also have insomnia and vice versa [23]. About 40% of patients in the study had a high risk of OSA. Another study showed that 50% of patients with ACS had moderate-to-severe OSA, with another reporting that 37% of hospitalized patients with ACD had insomnia and 27% were poor sleepers [23]. At least 70% of patients experienced moderate distress and fear of dying during ACS [23]. A Brazilian study showed OSA prevalence of 32.8% in an urban population [1]. Moderate-to-severe OSA is a very common pathology in MDD. In patients with OSA, the prevalence of depressive symptoms was as high as 63% and significantly reduced the quality of life [1]. Almost half of the patients with ACS have sleep disturbance and seems to be the most severe in the first few days post-ACS and decreases over the course of the next few months [20]. Almost 70% of patients have a moderate-to-intense fear of death and distress during ACS. Higher levels of distress are associated with an increased risk of hospitalization during cardiac events over a follow-up of three years [20]. The risk factors for sleep disorders are shown in Figure 1.

Comorbidities associated with sleep disorders

**Figure 2:**
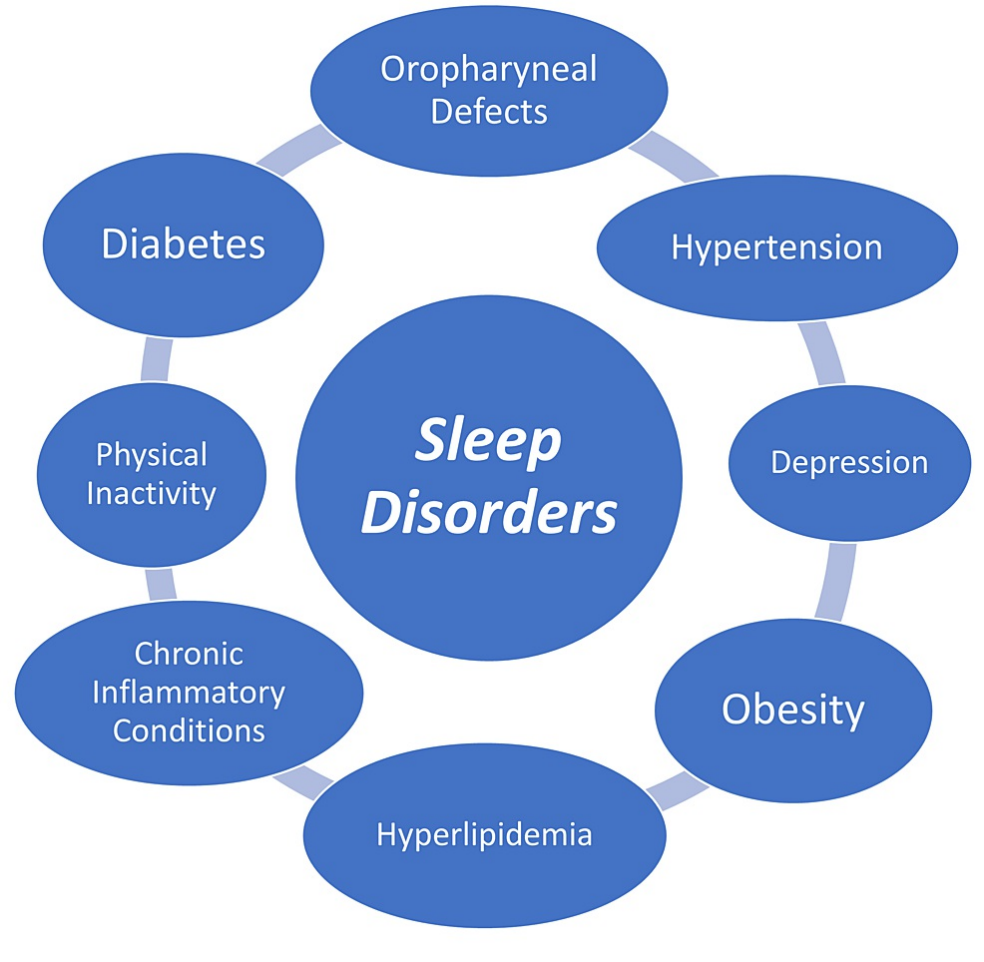
The comorbidities associated with sleep disorders.

Sleep-disordered breathing (SDB) is commonly associated with excessive daytime sleepiness (EDS) and increases the risk of incidence of CVD [24]. Smoking, male sex, oropharyngeal defects, and obesity are important risk factors for OSA [1]. OSA and SDB are important causes for chronic sleep fragmentation and sleep deficiency [1]. Modifiable risk factors for OSA include obesity, alcoholism, smoking, HTN, diabetes mellitus (DM), and hyperlipidemia. Factors like HTN and obesity have a bidirectional association with OSA [1]. There is a direct correlation between major risk factors for CVD like HTN, obesity, DM, hyperlipidemia, and physical inactivity with severity of OSA [1]. OSA causes intermittent nocturnal hypoxemia, persistently elevated sympathetic tone, and excessive daytime sleepiness [1]. There is a significant link between OSA risk and insomnia along with non-restorative sleep and frequent awakenings [23]. OSA is commonly associated with daytime sleepiness, fatigue, snoring, and breathing cessations during sleep [23]. OSA is associated with metabolic derangements like neuroendocrine dysfunction including hypertension, obesity, diabetes, dyslipidemia, and prothrombotic states are a progression of atherothrombotic illness [23]. There is a reciprocal link between inflammation and sleep disorders [23]. Intermittent hypoxia seems to have pro-angiogenic and anti-oxidant effects [23]. Sleep deprivation is shown to result in tissue-specific circadian cycle disruption leading to misalignment of the molecular clock in cardiomyocytes causing a disturbance in cellular/metabolic homeostasis making the cells more vulnerable to physiological stress [12]. Sleep deprivation has been shown to affect HRV following exercise [12]. Higher ESS, NOD, mean apnea duration, and ODI are observed in severe (AHI ≥30) OSA [1]. Higher homocysteine levels are associated with high prevalence of HTN in OSA [1]. An Australian study identified the association between gestational HTN and preeclampsia with OSA [1]. An American study showed an increased association between narcolepsy and glucose intolerance, peripheral neuropathy, and automobile-related accidents, as well as higher rates of OSA [1]. In the study, it was observed that EDS is associated with a higher risk of re-infarction than in patients without EDS, and it was established as an independent risk factor for adverse cardiac outcomes in post-MI patients [24]. Sleep disturbances along with fatigue were one of the most common symptoms affecting patients post-MI [25]. Sleep apnea and MD are independently associated with adverse cardiac outcomes post-MI [26]. Guidelines by the European Sleep Research Society recommend CBT as a first-line treatment for chronic insomnia. Pharmacotherapy may be considered for short-term treatment of insomnia (≤four weeks) [20]. The comorbidities associated with sleep disorders are shown in Figure 2.

Disturbed sleep and cardiac morbidity and mortality

Sleep disturbance has been associated with increased morbidity and mortality in MDD comorbid with CVD [27]. Sleep disturbance is an important symptom of depression and has a prevalence of 20-40% in patients with post-MI depression [20]. Insomnia and OSA are important issues associated with poor prognosis in CAD patients, and poor sleep is linked to higher risk of cardiac events [23]. Increased cardiac events are seen in patients with OSA and insomnia having difficulty initiating and maintaining sleep [23]. Day and night-time heart rate (HR) mean values were higher in post-MI depression, and higher night-time HR and depression are independent markers for survival in post-MI depression patients [28]. OSA is a serious life-threatening condition and increases CVD risk factors such as HTN, obesity, and diabetes leading to serious illnesses such as arrhythmias, CHF, and stroke [1]. Severe OSA is an important independent predictor for all-cause and cardiovascular mortality irrespective of ethnicity and race [1]. OSA increases the risk of HF by 140%, stroke by 60%, and CAD by 30% [1].The severity of AHI and cardiac risk showed a positive correlation proportional to the increase in AHI. The risk of cardiac events was 8.7% for normal AHI, and 19.7%, 27.8%, and 30.3% for patients with OSA of 5.1-14.9, 15-29.9, and ≥30, respectively, based on the ACC/AHA risk calculator [1]. In a study of middle-aged women with ACS, sleep quality index was predictive of recurrent cardiac events over a five-year follow-up [20]. In another study of 1,152 ACS patients, sleep disturbance was associated with higher all-cause mortality after 5-12 years [20]. According to another study, sleep disturbance post-ACS had a prevalence of 40-70% over a one-year follow-up [20]. In animal studies, selective serotonin reuptake inhibitors were effective in treating post-MI depression but had little effect on post-MI sleep disorders [29]. The efficacy of CBT is decreased after MI in patients with OSA [30]. Sleep disorders indicate a poor prognosis post-MI with double the risk of recurrent cardiac events and stroke [23]. In patients with insomnia post-MI, lack of restorative sleep was predictive of case fatality in the four weeks post-MI and 10-year risk of new cardiac events in women [23]. Three to six months after ACS in women, sleep quality index defined by symptoms of insomnia was predictive of recurrent cardiac events over the next five years [23]. A state of vital exhaustion associated with sleep disturbances in trouble falling asleep and repeated night-time awakenings along with profound fatigue after undergoing percutaneous coronary angioplasty (PCI) was predictive of new cardiac adverse events over a 1.5-year follow-up [23]. Mechanisms such as sympathetic overactivity, inflammation, oxidative stress, endothelial dysfunction, elevated cortisol, and hypercoagulability are associated with OSA and increase the risk of CVD and atherothrombotic events [23]. In OSA, sympathetic hyperactivity is associated with beta-2-adrenergic receptor desensitization leading to blunting of catecholamine-induced anti-inflammatory effects; therefore, these patients are unable to mount a proper stress response to ACS [23]. Acute MI patients with severe insomnia had significantly higher levels of fibrinogen linked to prothrombotic state increasing the risk for recurrent MI [23]. CVD patients with high OSA risk had increased vascular inflammation and faster progression of atherosclerosis [23]. Low cortisol levels at admission with acute MI were shown to predict early death in these patients [23]. Chronic intermittent hypoxia is an adaptive stimulus for myocardium in OSA [23]. According to a study, patients with severe OSA were associated with less severe cardiac injury in non-fatal ACSs due to ischemic preconditioning caused by chronic intermittent hypoxia [23]. A reduced sympathoadrenal response in ACS due to OSA may result in lower oxidative stress and myocardial damage [23].

**Figure 3:**
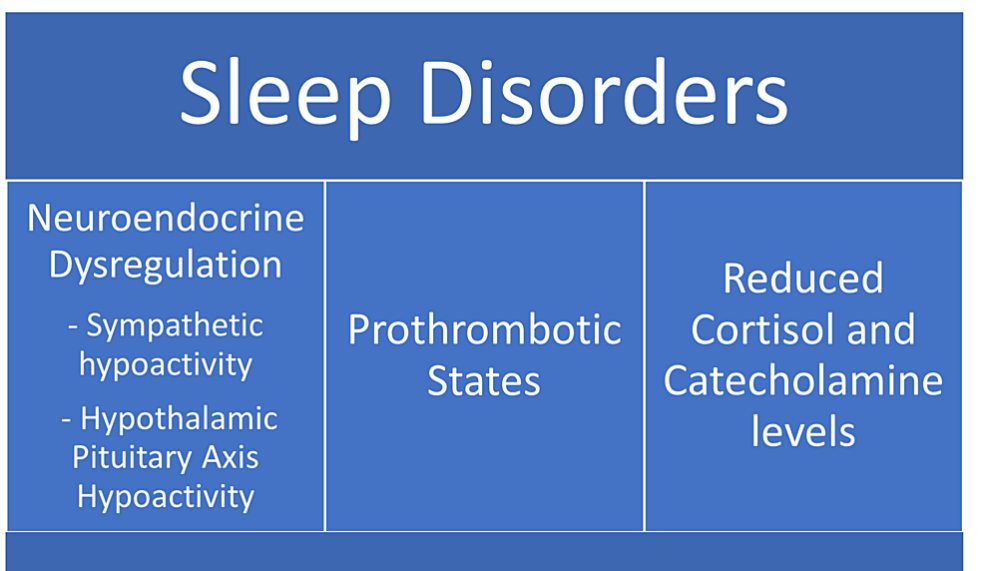
Mechanisms associated with cardiovascular disease morbidity and sleep disorders.

Cardiometabolic morbidity and activation of the stress system in patients with insomnia is closely linked with objective short sleep duration [23]. Overall, in patients with a high OSA risk and insomnia, the associated neuroendocrine dysregulation leads to sympathetic and hypothalamic-pituitary-adrenal axis hypoactivity and prothrombotic states lead to higher adverse outcomes post-MI, while lower levels of cortisol and catecholamines in patients with acute MI and high OSA risk without insomnia may potentially be cardioprotective due to chronic intermittent hypoxia [23]. SBD is a known cause of left atrial (LA) remodeling. Post-MI stress can substantially worsen healing process with concomitant SDB [31]. SBD is highly prevalent in patients with HF. SBD and OSA have multiple closely interconnected mechanisms [31]. SBD and OSA in combination with hypoxia increase sympathetic activity, BP, and myocardial oxygen demand predisposing to arrhythmias [31]. Hypoxia stimulates myocardial fibrosis in both acute and chronic HF [31]. SBD is a predictor of worse outcomes in CVD and impairs myocardial salvage in acute MI leading to adverse cardiac remodeling [31]. Atria are more susceptible to effects of SDB than ventricles due to thinner musculature [31]. LA function is an important marker for cardiac performance. LA dysfunction predisposes to atrial fibrillation, exacerbation of CHF, and increased mortality post-MI [31]. Compared to healthy individuals, echocardiogram (ECHO) analysis has shown that LA function is markedly reduced in patients with SDB [31]. LA dysfunction is associated with higher morbidity and mortality in the general population and HF patients. Reduced LA strain is linked to myocardial fibrofatty remodeling [31]. SBD severity correlated with reduced atrial function and had a close causal relationship over a three-month follow-up in patients with acute MI [31]. Atrial strain is a important marker of atrial dysfunction after MI in patients with SDB [31]. Ischemic damage during MI usually involves the ventricles, and atrial damage is mainly secondary to reduced ventricular function, sympathetic overactivity, and inflammation; however, recent studies have shown that atrial infarction may be concomitant in MI ranging from 0.7% to 42% [31]. The atrial damage in MI may be aggravated by presence of SDB both in acute and chronic setting [31]. Patients with severe SDB of Apnea-Hypoapnea Index (AHI) >15/hour had significantly impaired atrial function after acute MI [31]. SBD is associated with reduced LVEF and adverse cardiac remodeling. In patients with improving LVEF after MI, the severity of SDB was also reduced in a 12-week follow-up of patients post-MI [31]. Infarct size was also significantly large in patients with SDB [31]. OSA correlated with adverse cardiac remodeling post-MI [31]. NT-pro-brain natriuretic peptide (BNP) levels were elevated in patients with SDB and atrial dysfunction at baseline, and SDB was associated with reduced LVEF recovery following MI. A shift was observed in the prevalence of AHI >15/hour in patients post-MI from 60% initially to 40% at follow-up suggesting AHI measured at initial hospitalization may be an overestimation [31]. LA dysfunction is associated with SDB both acutely following MI and chronically [31]. Regular high-intensity exercise may promote atherosclerotic changes and cause subclinical myocardial damage [12]. Recurrent sleep restriction is shown to increase the cardiac stress response related to acute high-intensity exercise in healthy individuals [12]. Studies have shown that chronic short duration sleep coupled with intense physical exercise increased the risk of cardiac events [12]. Cardiac troponin release due to exercise is a physiologic myocardial stress response due to impact on cellular inflammation and autonomic system, sleep deprivation may lower the threshold of myocardial stress [12]. Cardiac troponin response to exercise is predictive of increased mortality and future risk of cardiac events [12]. A Bulgarian study showed that 36.6% of patients with sleep apnea had associated pericardial effusion [1]. Sleep disorders are linked to poor prognosis in patients post-MI [20]. Poor weekly sleep quality is predictive of mortality within 28 days for men post-MI, and in women sleep disturbances are associated with an increased 10-year risk of cardiac events [20]. It is evident that sleep disturbance results in poor prognosis in ACS patients [20]. Sleep disturbance leads to poor prognosis in patients post-ACS [20]. Behavioral interventions for sleep improvement to improve prognosis in ACS is needed [20]. CBT for insomnia can be tailored to patients post-MI [20]. Cardiac nurses should be trained to observe symptoms of sleep disturbance, fatigue, and anxiety in patients post-MI in a hospital setting [28]. It is crucial to screen patients for the risk of OSA and SDB in cardiovascular population with polysomnography. ECG and AHI are essential tools to identify patients for screening, Treatments for OSA such as automatic positive airway pressure have shown to improve LVEF in HF patients [31]. The mechanisms associated with CVD morbidity and Sleep disorders are shown in Figure 3.

## Conclusions

Based on our research data, sleep disorders play a significant role in determining the prognosis of patients post-MI. In clinical practice, the majority of patients with heart disease are not effectively screened for sleep disorders. Proactive screening and targeted treatment for sleep disorders in patients with CVD is a highly effective way to reduce the overall morbidity in patients post-MI as many of the mechanisms and factors linked to the causation of sleep disorders are also associated with the causation of co-morbidities commonly seen in CVDs such as obesity, diabetes, MD, HTN. Both psychotherapy and pharmacotherapy are effective for sleep disorders in patients with CVD. Medical professionals should be trained to be vigilant in screening all patients post-MI. We encourage large-scale observational studies and prospective and retrospective cohort studies regarding the prevalence and significance of sleep disorders in the prognosis of patients post-MI.
